# Supplementary material for: Single-Nucleus Chromatin Accessibility and Epigenetic Study Uncover Cell States and Transcriptional Regulation of Epidermis in Hidradenitis Suppurativa
Source: Biomedicines. 2025 Jun 30;13(7):1599. doi: 10.3390/biomedicines13071599 (PMC12292286; doi:10.3390/biomedicines13071599)
Supplement: Supplementary file 1 [file biomedicines-13-01599-s001.zip › fig1&2.pptx]

## Slide 1
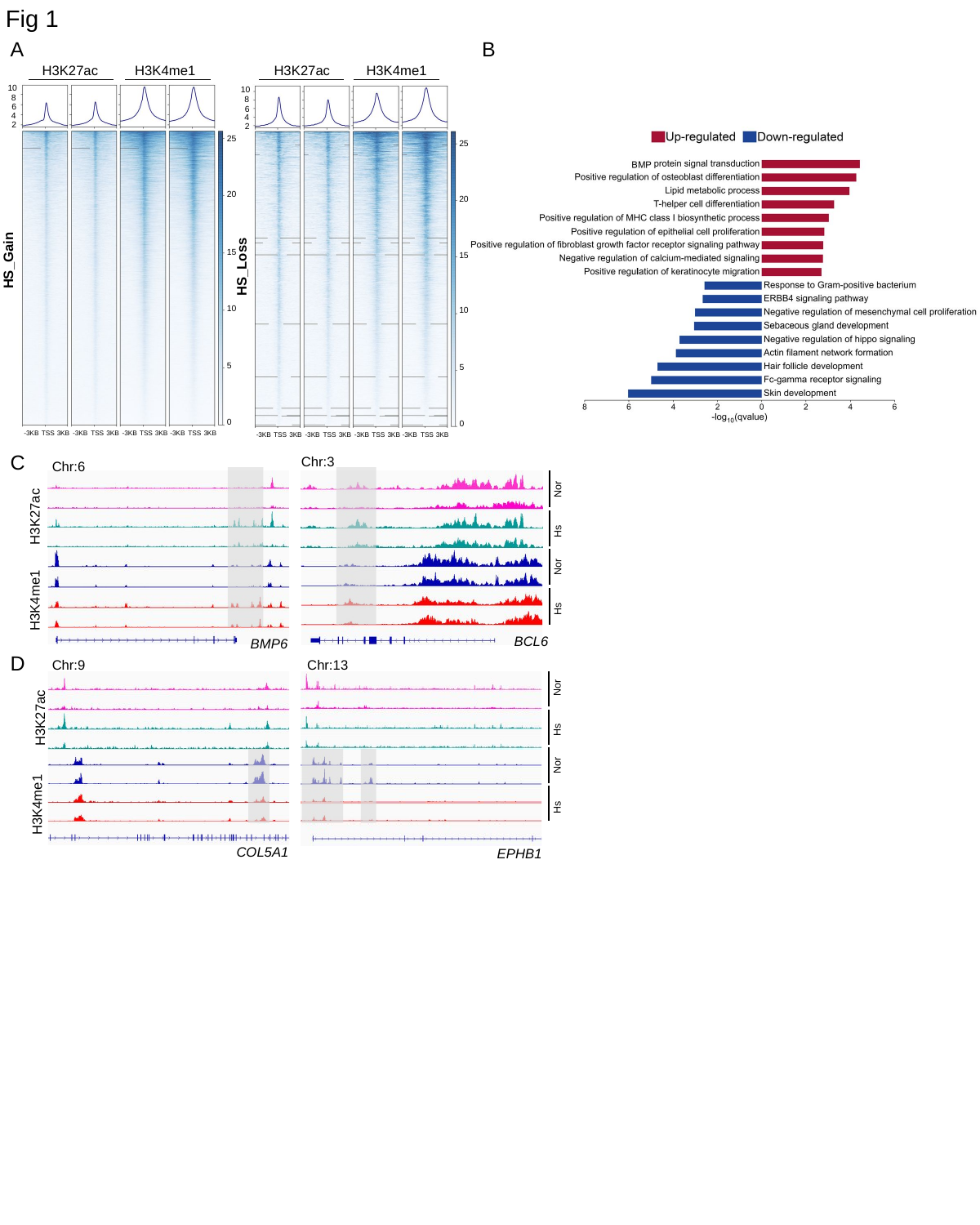

Fig 1
A
B
H3K27ac
H3K4me1
10
8
6
4
2
25
20
HS_Gain
15
10
5
0
-3KB
TSS
3KB
-3KB
TSS
3KB
-3KB
TSS
3KB
-3KB
TSS
3KB
H3K27ac
H3K4me1
10
8
6
4
2
25
20
HS_Loss
15
10
5
0
-3KB
TSS
3KB
-3KB
TSS
3KB
-3KB
TSS
3KB
-3KB
TSS
3KB
C
Chr:3
Chr:6
Nor
H3K27ac
Hs
Nor
H3K4me1
Hs
BCL6
BMP6
D
Chr:9
Chr:13
Nor
H3K27ac
Hs
Nor
H3K4me1
Hs
COL5A1
EPHB1

## Slide 2
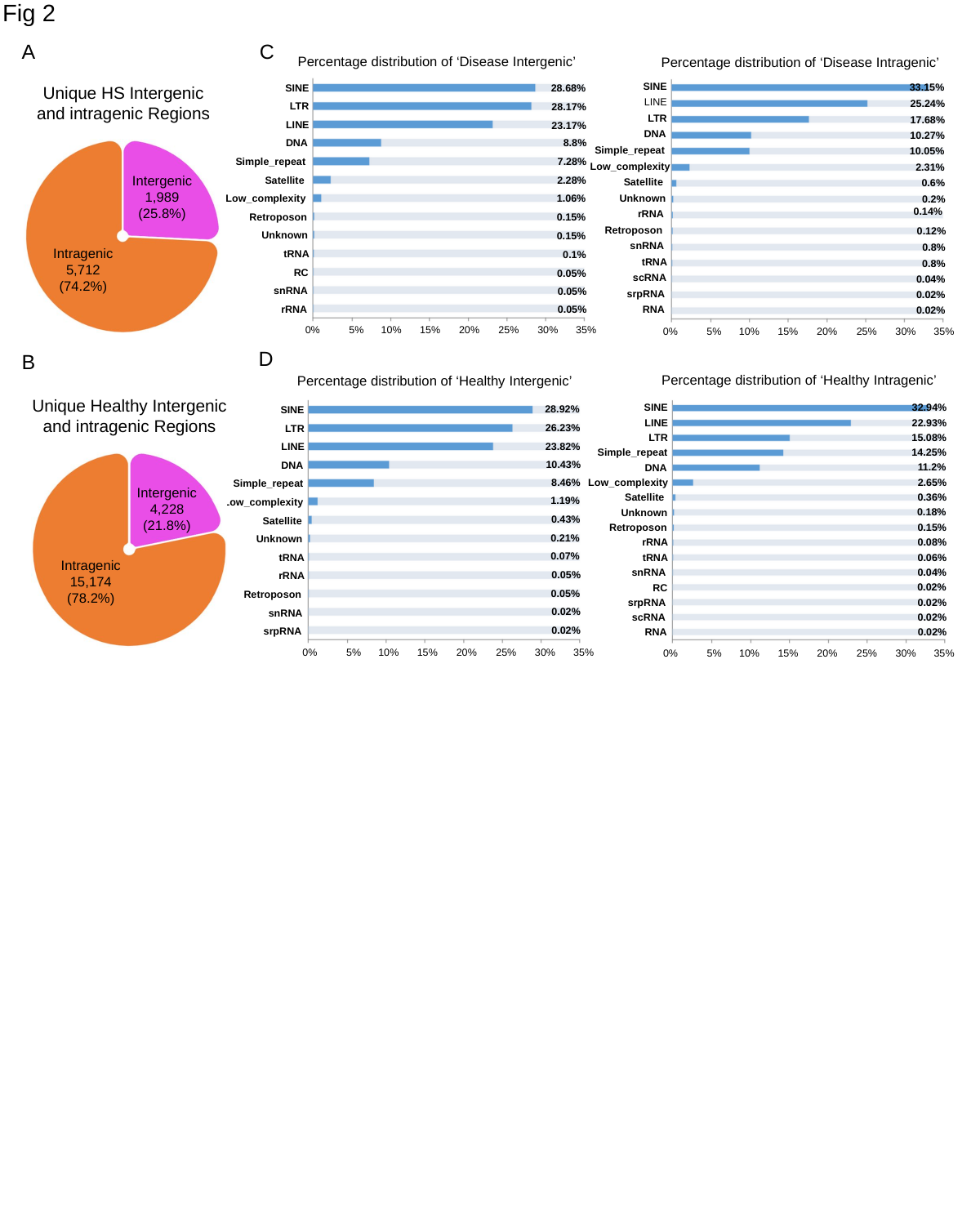

Fig 2
A
C
Percentage distribution of ‘Disease Intergenic’
SINE
28.68%
LTR
28.17%
LINE
23.17%
8.8%
DNA
Simple_repeat
7.28%
Satellite
2.28%
Low_complexity
1.06%
Retroposon
0.15%
Unknown
0.15%
tRNA
0.1%
RC
0.05%
snRNA
0.05%
rRNA
0.05%
0%
5%
10%
15%
20%
25%
30%
35%
Percentage distribution of ‘Disease Intragenic’
SINE
33.15%
LINE
25.24%
LTR
17.68%
DNA
10.27%
Simple_repeat
10.05%
Low_complexity
2.31%
Satellite
0.6%
Unknown
0.2%
0.14%
rRNA
Retroposon
0.12%
snRNA
0.8%
tRNA
0.8%
scRNA
0.04%
srpRNA
0.02%
RNA
0.02%
0%
5%
10%
15%
20%
25%
30%
35%
Unique HS Intergenic and intragenic Regions
Intergenic
1,989
(25.8%)
Intragenic
5,712
(74.2%)
D
B
Percentage distribution of ‘Healthy Intragenic’
32.94%
SINE
22.93%
LINE
LTR
15.08%
14.25%
Simple_repeat
11.2%
DNA
2.65%
Low_complexity
0.36%
Satellite
0.18%
Unknown
0.15%
Retroposon
0.08%
rRNA
0.06%
tRNA
0.04%
snRNA
0.02%
RC
0.02%
srpRNA
0.02%
scRNA
0.02%
RNA
0%
5%
10%
15%
20%
25%
30%
35%
Percentage distribution of ‘Healthy Intergenic’
28.92%
SINE
26.23%
LTR
LINE
23.82%
10.43%
DNA
8.46%
Simple_repeat
1.19%
Low_complexity
0.43%
Satellite
0.21%
Unknown
0.07%
tRNA
0.05%
rRNA
0.05%
Retroposon
0.02%
snRNA
0.02%
srpRNA
0%
5%
10%
15%
20%
25%
30%
35%
Unique Healthy Intergenic and intragenic Regions
Intergenic
4,228
(21.8%)
Intragenic
15,174
(78.2%)
