# Supplementary material for: Single-Nucleus Chromatin Accessibility and Epigenetic Study Uncover Cell States and Transcriptional Regulation of Epidermis in Hidradenitis Suppurativa
Source: Biomedicines. 2025 Jun 30;13(7):1599. doi: 10.3390/biomedicines13071599 (PMC12292286; doi:10.3390/biomedicines13071599)
Supplement: Supplementary file 1 [file biomedicines-13-01599-s001.zip › Supplementary caption.pdf]

Supplementary caption:

Supplementary Figure 1. High-resolution confocal images of ATF3 expression in healthy and HS lesional skin. (A) and (B) Representative images showing the expression of KRT14(green) and ATF3 (red), along with DAPI (blue) in healthy skin (A) and HS skin lesions (B). The experiment was repeated on 5 individuals for healthy and HS skin, respectively. Scale bars, 50 μm.

Supplementary Table 1: Description of the patient information and their respective sample for various experiments included in this study.

Supplementary Table 2: The information of HS-associated gene loci derived from snATAC-seq.
