# Supplementary material for: Single-Nucleus Chromatin Accessibility and Epigenetic Study Uncover Cell States and Transcriptional Regulation of Epidermis in Hidradenitis Suppurativa
Source: Biomedicines. 2025 Jun 30;13(7):1599. doi: 10.3390/biomedicines13071599 (PMC12292286; doi:10.3390/biomedicines13071599)
Supplement: Supplementary file 1 [file biomedicines-13-01599-s001.zip › supplementary Table S1.pdf]

Supplementary Table S1. Description of the patient information and their respecitve sample for various experiments included in this study.

Condition	Gender	Race	Body site	Experiments
Healthy 1	F	Caucasian	Breast	snATAC,IF
Healthy 2	F	Black	Breast	snATAC,IF
Healthy 3	F	Caucasian	Breast	IF
Healthy 4	F	Caucasian	Breast	IF
Healthy 5	M	Black	Abdomen	IF
HS 1	M	Black	Axilla	snATAC,IF
HS 2	F	Black	Inframammary	snATAC,IF
HS 3	F	Black	Axilla	IF
HS 4	M	Black	Axilla	IF
HS 5	F	Black	Axilla	IF

Abbreviation: snATAC: snATAC-sequence; IF:immunofluorsence

Note: HS patients above mentioned were Hurley late stage II/III and failure to drug treatment before surgery.
