# Supplementary figures and images for: Single-Nucleus Chromatin Accessibility and Epigenetic Study Uncover Cell States and Transcriptional Regulation of Epidermis in Hidradenitis Suppurativa
Source: Biomedicines. 2025 Jun 30;13(7):1599. doi: 10.3390/biomedicines13071599 (PMC12292286; doi:10.3390/biomedicines13071599)

## Slide 1
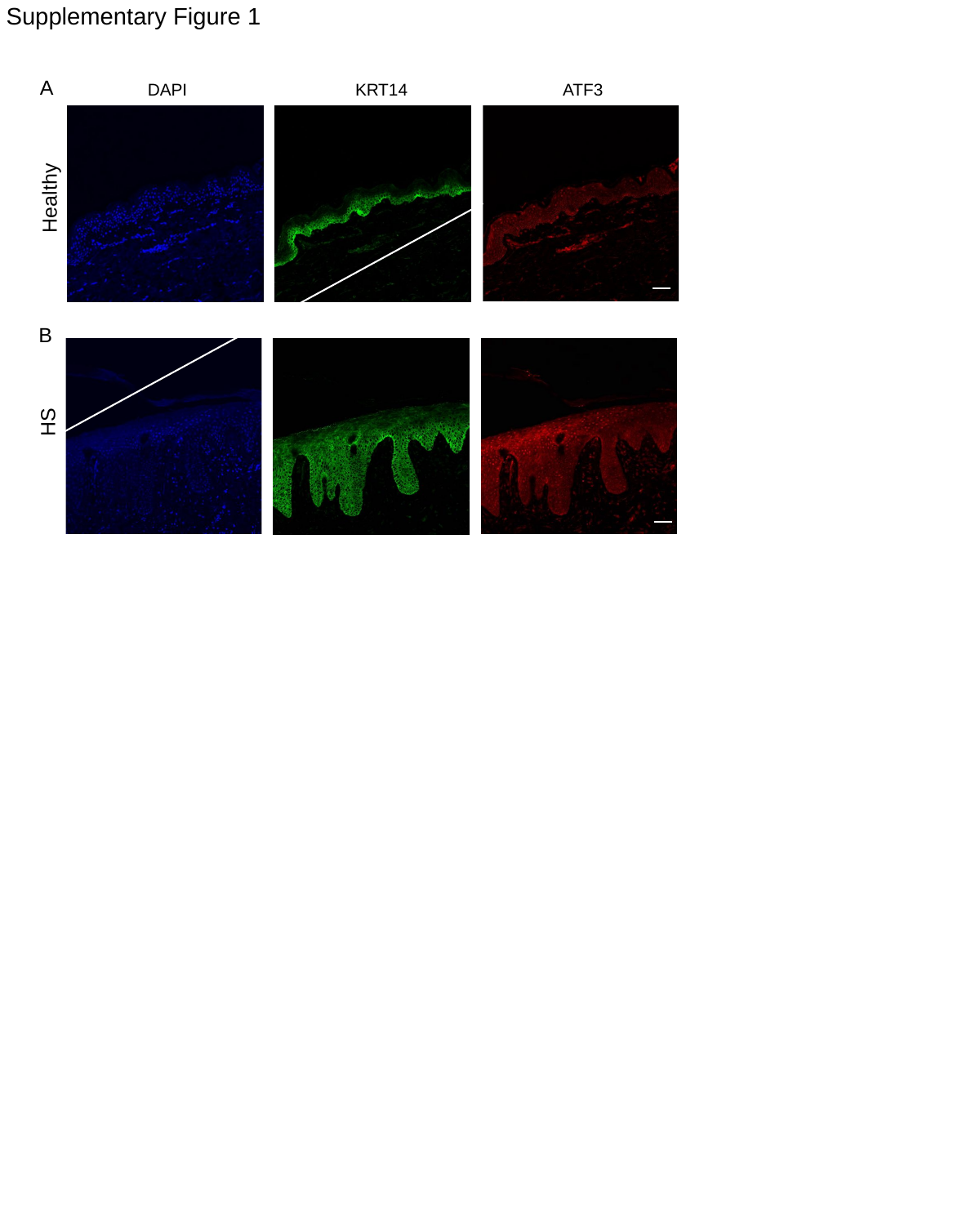

Supplementary Figure 1
A
KRT14
ATF3
DAPI
Healthy
B
HS

Supplement: Supplementary file 1 [file biomedicines-13-01599-s001.zip › Supplementary Figure .pptx]
